# Editorial: Role of mitochondrial stress response in metabolic health

**DOI:** 10.3389/fendo.2024.1504718

**Published:** 2024-10-17

**Authors:** Renata O. Pereira, Susanne Keipert

**Affiliations:** ^1^ Fraternal Order of Eagles Diabetes Research Center and Division of Endocrinology and Metabolism, Roy J. and Lucille A. Carver College of Medicine, University of Iowa, Iowa City, IA, United States; ^2^ Department of Molecular Biosciences, The Wenner-Gren Institute, Stockholm University, Stockholm, Sweden

**Keywords:** mitochondria, mitohormesis, integrated stress response (ISR), mitokine, metabolic health, GDF15, FGF21, MOTS-c

Mitochondria are multifaceted organelles with a complex metabolic machinery that mediates the conversion of multiple metabolic substrates to ATP, thereby providing energy for most cellular functions. In addition to their essential role in metabolism, mitochondria also regulate cell death pathways, buffer intracellular calcium, and serve as signaling hubs in the cell. To support all these different functions, mitochondria are highly dynamic and adaptable, and are crucial for stress response activation to restore homeostasis (1). A variety of endogenous and exogenous stressors are known to affect mitochondrial function, leading to changes in oxidative metabolism or redox homeostasis. Interestingly, those changes are not only associated with metabolic diseases but are also thought to be protective. In the past decade, several studies demonstrated that mitochondrial dysfunction of different etiologies can induce cell-autonomous and cell non-autonomous pathways such as the integrated stress response (ISR) and the mitochondrial unfolded protein response (UPR^mt^) aimed at restoring cellular and organismal homeostasis (2, 3). These mitohormetic responses ultimately lead to improved systemic metabolic health by mechanisms that often involve changes in the antioxidant defense system or the secretion of endocrine acting peptides. The resulting positive metabolic effects often include improvements in energy and substrate homeostasis, retarding the development of obesity, insulin resistance or other aging associated diseases. The molecular signals activating the stress response pathways as well as the mechanisms of action mediating the adaptive responses are still incompletely understood. Expanding the understanding of the mitochondrial stress response and its role in regulating metabolic health might lead to the discovery of novel therapeutic avenues for the treatment of metabolic diseases.

This Research Topic highlights pathways and molecules induced in response to mitochondrial stress that regulate inter-organ communication and metabolic fitness, leading to enhanced phenotypes. Here, we compiled a series of original research articles, and reviews that amplify our understanding of the mitochondrial stress response, and how it can be leveraged to improve systemic metabolic health.

Fibroblast growth factor 21 (FGF21) and Growth differentiation factor 15 (GDF15) are recognized as stress-responsive cytokines that can regulate energy balance by increasing energy expenditure or suppressing food intake, respectively. Endogenous induction of FGF21 and GDF15 in mouse models of mitochondrial disturbances has been shown to improve systemic metabolic health (4–8). Jena et al. reviewed the roles of FGF21 and GDF15 on metabolism and highlighted several studies on transgenic mouse models of mitochondrial stress, dissecting the individual and potential synergistic roles played by FGF21 and GDF15 and its effects on systemic metabolic adaptations, including browning of white adipose tissue, improvements in glucose homeostasis and resistance to diet-induced obesity. In this mini review, one of the reported mouse models of mitochondrial stress was the mUcp1-transgenic mouse, which ectopically expresses uncoupling protein 1 (UCP1) in skeletal muscle (6, 7). The original research article by Gil et al. used this mouse model and performed a comprehensive 24-hour muscle and plasma profiling of male and female animals, to evaluate changes in the temporal signatures of the cell-autonomous and endocrine responses observed in mUcp1-transgenic mice. The authors described a progressive increase in the muscle ISR during their active phase, with a subsequent peak in circulating levels of the myokines FGF21 and GDF15 in the early resting phase. Subsequently, increased antioxidant capacity and ferritin levels were proposed to counteract excessive iron-dependent lipid peroxidation and ferroptosis. This work provides a detailed temporal dynamics analysis of the ISR activation in skeletal muscle in a relevant model of mitochondrial stress along with its downstream endocrine mediators of metabolic health. Further, the reported findings highlight the need to consider diurnal variations in sampling for diagnostic biomarker analysis.

Another relevant mouse model of mitochondrial stress in skeletal muscle is the OPA1 muscle-specific knockout (KO) mouse, where FGF21 is induced as a myokine leading to improved systemic metabolic health (9). There are accumulated reports demonstrating that activating transcription factor 4 (ATF4), the main effector of the ISR, is an important regulator of increased expression of FGF21 upon cellular stress (4). In this Research Topic, Streeter et al. crossed the muscle specific OPA1 KO mouse with a muscle-selective ATF4 KO mouse (OPA1/ATF4 DKO mice) to test the requirement of ATF4 for FGF21 induction and associated metabolic benefits. FGF21 levels were reduced in DKO males, indicative of a significant role for ATF4 in FGF21 induction. Conversely, female DKO mice showed an induction of circulating FGF21 levels, highlighting an important sex-dimorphism in this model in regards to FGF21 regulation and suggesting that additional factors are likely involved in regulating FGF21 levels in the muscle of OPA1 KO mice. Interestingly, this sex dimorphism in FGF21 circulating levels had no effect on glucose tolerance, insulin sensitivity as well as protection from weight gain, which were similar between male and female OPA1 and OPA1/ATF4 DKO mice. The authors suggested that changes in GDF15 could play a role in mediating metabolic adaptations, in situations where FGF21 induction is reduced. In future studies, it will be important to identify additional pathways regulating FGF21 and GDF15 induction in response to mitochondrial stress, as well as to dissect the independent and potentially synergistic roles of endogenous FGF21 and GDF15 in health and disease in both male and female mice.

In addition to FGF21 and GDF15, other peptides have been suggested to act as mitokines, mediating improvements in metabolic health. Mitochondria have a unique circular genome traditionally thought to encode only 13 protein-coding genes. However, recently, short open reading frames within the mitochondrial genome have been discovered, which give rise to bioactive peptides actively regulating metabolism. One such peptide, MOTS-c, a 16-amino acid derived from the 12S rRNA, is linked to metabolic homeostasis and anti-inflammatory effects. The review by Zheng et al., discussed the discovery and physiological role of MOTS-c, in addition to its potential for the treatment of various pathological conditions, such as cardiovascular disease, insulin resistance and inflammation. They also provided additional insights into the molecular mechanisms mediating MOTS-c functions, which could inform future research, including the development of therapeutics based on activation of MOTS-c secretion as a mitokine.

Regarding MOTS-c, Li et al. suggested a novel, muscle-type specific role to improve metabolism. This original research article investigated changes in UPR^mt^ in skeletal muscle of insulin-resistant male Wistar rats fed a high fat diet (HFD). The authors documented that fast-twitch Tibialis Anterior (TA) muscle exhibits decreased glucose transporter expression and impaired mitochondrial function after HFD, while slow-twitch Soleus muscle is mostly unaffected. The authors attributed the mechanisms of this differential response to HFD to increased UPR^mt^ activation, leading to higher MOTS-c expression and improved mitochondrial respiratory function in the Soleus muscle relative to TA muscle. The authors concluded that the Soleus muscle is more resistant to developing diet-induced impairments in glucose uptake and mitochondrial function due to upregulation of UPR^mt^ proteins, including induction of MOTS-c.

Taken together, all studies in this Research Topic contribute to our understanding of downstream regulators of the mitochondrial stress response, from transcription factors induced by different stress response pathways to regulatory mitokines that mediate systemic metabolic adaptations to maintain homeostasis and improve health. In the studies published herein, detailed analysis of temporal, sex-specific and tissue-specific dynamics related to mitochondrial stress shed light on the complexity of these adaptive mechanisms. Further research is needed to fully uncover the mechanisms underlying the beneficial effects of the mitochondrial stress response on metabolism, including identification of novel mitokines, its regulators and their pleiotropic effects in health and disease.
